# Uretero-Ureterostomy Combined With Unilateral Nephrostomy as a Method of Urinary Diversion Following Radical Cystectomy

**DOI:** 10.7759/cureus.27501

**Published:** 2022-07-31

**Authors:** Christos Papadimitriou, Charalampos Deliveliotis, Athanasios Dellis, Wilfried Martin, Iraklis Mitsogiannis

**Affiliations:** 1 Department of Urology, St. Antonius Hospital Gronau, Gronau, DEU; 2 2nd Department of Urology, Sismanoglio Hospital, National and Kapodistrian University of Athens, Athens, GRC; 3 1st Department of Urology, National and Kapodistrian University of Athens, Athens, GRC; 4 Department of Urology, Agaplesion General Hospital Hagen, Hagen, DEU

**Keywords:** ureteroureterostomy, urosepsis, ureterohydronephrosis, renal function, bladder cancer

## Abstract

Introduction

Uretero-ureterostomy combined with unilateral nephrostomy is a rarely performed urinary diversion following radical cystectomy for muscle-invasive bladder cancer. The aim of this study is to assess the efficacy and safety of the procedure.

Materials and methods

Patients with muscle-invasive bladder cancer and poor performance status were enrolled in this retrospective, observational, single-centre study, carried out between December 2018 and November 2020. The patient's renal function was regularly assessed with serum creatinine and estimated glomerular filtration rate (eGFR). Evaluation of peri- and postoperative complications was performed based on clinical, laboratory, endoscopic, ultrasound and other radiological studies findings. The patient’s status was assessed for 12 months.

Results

Thirty-six patients with a mean age of 77.4±8.6 years were enrolled in the study. 86.11% of patients had an American Society of Anesthesiologists Score ≥3 and 91.66% had an age-adjusted Charlson comorbidity index of ≥6. Slight deterioration of renal function, although not statistically significant, was observed. 36.11% of the patients developed permanent unilateral pelvic dilatation. Acute pyelonephritis, urosepsis, pyonephrosis and anastomotic leak were observed in 22.22%, 8.33%, 5.55% and 5.55% of patients, respectively; all were treated either conservatively and/or with minimally invasive procedures (nephrostomy, JJ-stent insertion) without any need for open surgical revision.

Conclusions

Ureteroureterostomy combined with unilateral nephrostomy is a safe and effective method of urinary diversion following radical cystectomy for muscle-invasive bladder cancer with easily manageable complications.

## Introduction

Bladder cancer (BC), which is the 10th most common newly diagnosed malignancy, ranks 13th in deaths worldwide [1]. Similarly, BC is the fourth most frequently diagnosed malignancy in men and the 12th in women and ranks 10th in men and 15th in women in deaths in Germany [2].

In patients with muscle invasive, as well as in those with BCG-refractory non-muscle-invasive BC, radical cystectomy is recommended [3,4], with a non-continent diversion without the use of the small intestine (usually uretero-cutaneostomy) being indicated in high-risk patients [5-8]. Alternatively, urine is diverted via uretero-ureterostomy through a unilateral nephrostomy and, rarely, via uretero-ureterocutaneostomy [5,6,9].

The first reference to uretero-ureterostomy was made in 1961 by Arnold et al. in the USA [10]. While the procedure has been subsequently modified and small-scale clinical and animal experimental reports describe satisfactory functionality, uretero-ureterostomy with unilateral nephrostomy is not a widely used method of urinary diversion [10-15]. The aim of this clinical trial was to retrospectively evaluate the peri- and postoperative course of patients undergoing uretero-ureterostomy with unilateral nephrostomy.

## Materials and methods

Patients with stage T2-4b N0/N1 M0/M muscle-invasive BC, without osseous metastases, being at high surgical or anaesthesiologic risk and/or low life expectancy scheduled for radical cystectomy were included in the present study, which took place in the Department of Urology in General Hospital of Hagen in Germany. Exclusion criteria were a solitary kidney (congenital, acquired), bone metastases, haemodialysis, stricture of the upper third of the ureter, bilateral staghorn calculi, retroperitoneal fibrosis and previous ureterolysis. All surgical procedures were performed under general anaesthesia by two experienced urological surgeons. Ceftriaxone was administered intravenously as chemoprophylaxis. Preoperatively, an ultrasound-guided percutaneous nephrostomy tube 14-16 F was placed. After excision of the bladder, both ureters were developed cranially up to the level of the common iliac artery, where one ureter (usually the left) was transferred contralaterally, through a tunnel under the root of the mesentery, to be anastomosed with the other in an end-to-end fashion, using 5-0 absorbable interrupted sutures. The anastomosis was carried out over a 7F 40cm JJ catheter, which was inserted over a hydrophilic guidewire in both renal pelves. After the JJ stent is removed percutaneously under sedoanalgesia in the sixth postoperative week under pre-interventional, targeted antibiotic prophylaxis via the existing nephrostomy tube channel using a nephroscope, the nephrostomy tube is changed.

Absolute success was defined as the survival of the patient without anastomosis complications requiring open surgical revision with the non-fatal complication of anastomosis and eGFR reduction of less than 25%. Qualified success was defined as the survival of the patient without anastomosis complications requiring open surgical revision with the non-fatal complication of anastomosis.

Follow-up included scheduled visits to Clinic after six weeks and at the third, sixth, ninth and 12th postoperative months. Clinical examination, laboratory studies and sonographic and radiological studies were carried out during the visits. Renal function was assessed using serum creatinine and estimated glomerular filtration rate (eGFR). The ureteric stent was removed at the sixth postoperative week, whereas replacement of the nephrostomy tube was carried out every six weeks, under fluoroscopic guidance.

All patients signed an informed consent form for surgery. The tenets of the Declaration of Helsinki were fully respected. The study was approved by the local Institutional Review Board Committee.

Statistical analysis was carried out with IBM Statistical Package for the Social Sciences (SPSS) statistics version 22. We were able to examine with an independent sample t-test. The normality of the data was assessed using a histogram as a graphical method. Because the examined parameters did not follow a normal distribution, we used also the nonparametric Wilcoxon sign rank test. Statistical significance was set at 5% (p=0.05). In addition, we performed a Kaplan Meier survival analysis. An a-priori power analysis using G*Power 3 [16] to test the difference from constant in one sample using an effect size of d = 0.80 and an alpha of 0.05 showed that 19 participants were required to achieve a power of 0.95. The values are presented in terms of the sample mean and the standard deviations of the sample.

## Results

Thirty-six patients (26 males and 10 females) with a mean age of 77.4±8.6 years were included in the study. Thirty-one patients (86.11%) had an American Society of Anaesthesiologists Score ≥3 (ASA-score ≥ 3), whereas 33 (91.66%) had an age-adjusted Charlson comorbidity index ≥ 6. Patients were followed for a mean of 14.73±8.2 months. We herein present our data at the 12-month follow-up. Two patients were excluded from subsequent follow-up in the third and ninth month because of the insertion of a nephrostomy tube in the contralateral kidney due to complications of the anastomosis. The previous history of urothelial cancer, smoking, chronic exposure to hazardous substances in the workplace and pelvic radiation were risk factors for BC in 13, 29, seven and six patients, respectively. Patients’ demographics and type of surgery for BC are presented in Table 1.

**Table 1:** Demographic data of the patients included in the study ASA-score: American Society of Anesthesiologists-Score, eGFR: estimated glomerular filtration rate, No.: number, SD: standard deviation

Demographic Data	
Sex	
No. of patients	36
No. of female patients	10
No. of male patients	26
Age	
Min	56
Max	90
Average	77.44
SD	8.58
Baseline Creatinine (mg/dl)	1.44±0.85
Baseline eGFR (ml/min)	54.03±22.85
Baseline Urea (mg/dl)	45.92±24.17
Combination of the cystectomy with:	
Lymphadenectomy	15
Partial ileectomy	1
Rectal resection with permanent colostomy	2
Rectosigmoidectomy with permanent colostomy	1
Partial Sigmoidectomy	1
Abdominal hernia repair with mesh location	1
Urethrectomy	1
Colpectomy	1
Type of carcinoma	
Transitional cell carcinoma	33
Squamous cell carcinoma	2
Sarcomatoid carcinoma	1
Tumor Grade (T)	
T2	13
T3	14
T4	9
Metastatic lymph node Grade (N)	
Nx	21
N0	8
N1	3
N2	4
ASA - Score	
2	5
3	26
4	5
Age-adjusted Charlson comorbidity index	
3	1
4	1
5	1
6	15
7	10
8	3
9	2
10	0
11	3

**Figure 1:**
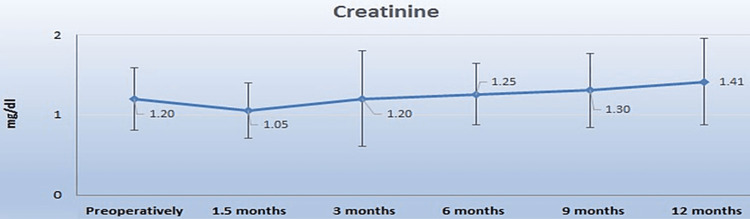
Graph of the mean creatinine curve (mg/dl) with the standard deviation up to 12 months

**Figure 2:**
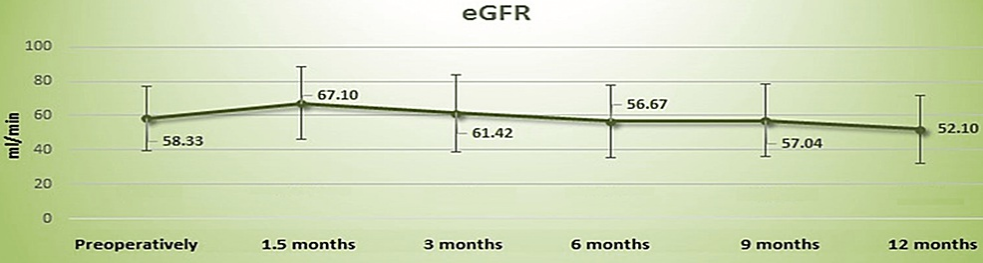
Graph of the mean eGFR (mL/min) with the standard deviation up to 12 months

Renal function was found to slightly deteriorate over the follow up period, although not significantly. Overall, mean serum creatinine and eGFR changed from 1.20±0.39 mg/dL and 58.33±18.88 mL/min preoperatively to 1.25±0.39 mg/dL (p=0.30) and 56.67±21.05 mL/min (p=0.38) after six months and 1.41±0.54 mg/dL (p=0.051) and 52.10±19.64 mL/min (p=0.13) after 12 months (Figures 1, 2). A similar slight deterioration of renal function was noticed in the 13 patients with pelvic dilatation; mean serum creatinine increased from 1.15±0.40 mg/dL preoperatively to 1.24±0.46 mg/dL after six months (p=0.33) and 1.47±0.46 mg/dL after 12 months (p=0.09), whereas eGFR decreased from a preoperative 61.27±23.83 mL/min to 58.81±23.77 mL/min at the six-month (p=0.41) and 49.90±21.21 mL/min (p=0.18) at the 12-month follow-up. Change in renal function during the 12-month follow-up did not differ significantly between patients with and without pelvic dilatation (Table 2).

 

**Table 2:** Renal function in patients with and without unilateral pelvic dilatation

Course of renal function									
	Preoperatively			6 months			12 months		
	Patients with unilateral pelvic dilatation	Patients without unilateral pelvic dilatation	P-Value	Patients with unilateral pelvic dilatation	Patients without unilateral pelvic dilatation	P-Value	Patients with unilateral pelvic dilatation	Patients without unilateral pelvic dilatation	p-Value
eGFR	61.3±23.8	56.7±15.1	0.3	58.8±23.8	55.3±18.9	0.37	49.9±21.2	53.3±18.6	0.37
Creatinine	1.2±0.4	1.2±0.4	0.30	1.2±0.5	1.3±0.3	0.45	1.5±0.5	1.4±0.6	0.36

Complications, occurring before and after the 15th postoperative day, were either nephrostomy-related or uretero-ureterostomy-related (Tables 3, 4). Leakage from the anastomosis was noticed in two cases (one primary and the other one following subsequent bowel operation) and was dealt with accordingly with insertion of a contralateral nephrostomy tube and simultaneous nephroscopic JJ-catheter implantation in the case with the secondary leakage (Table 4). Two patients died because of complications of cystectomy on the 15th and 22nd postoperative days.

**Table 3:** Nephrostomy-related complications

Complications of nephrostomy		
Time of the complication	Type of complication	Number of patients
Intraoperative	Inability to place the nephrostomy	1
	Placement of nephrostomy after retrograde dilatation of the pelvis through ureteric catheter	2
	Inability to remove percutaneously the JJ-Stent	1
	Removal of JJ-Stent with placement of 2^nd^ nephrostomy on the contralateral side	1
	Bleeding	2
	Hematoma	1
	Perforation of the renal pelvis	2
	Bowel perforation	1
Early postoperative (˂15 days)	Dislodgement	2
	Inability to change the nephrostomy	2
	Reinsertion of the nephrostomy on the ipsilateral side	2
	Reinsertion of the nephrostomy on the contralateral side	1
	Obstruction	1
	Bacteriuria	36
	Acute pyelonephritis	1
Late postoperative (≥15 days)	Partial displacement	2
	Dislodgement	10
	Inability to change the nephrostomy	2
	Reinsertion of the nephrostomy on the ipsilateral side	2
	Obstruction	4
	Acute renal failure	5
	Acute pyelonephritis	5
	Pyonephrosis	2
	Urosepsis	3
	Bacteriuria	36
	Hematuria	3

**Table 4:** Complications of uretero-ureterostomy

Complications of uretero-ureterostomy		
Time of the complication	Type of complication	Number of patients
Intraoperative	None	0
Early postoperative (˂15 days)	Acute pyelonephritis	2
Late postoperative (≥15 days)	Insufficiency	1
	Intraoperative anastomosis injury with insufficiency during ileus surgery	1
	Urinoma	2
	Placement of a 2nd nephrostomy at the contralateral site	2
	Placement of new JJ-Stent	1
	Ureterohydronephrosis Grade 1 of the contralateral kidney	13

**Figure 3:**
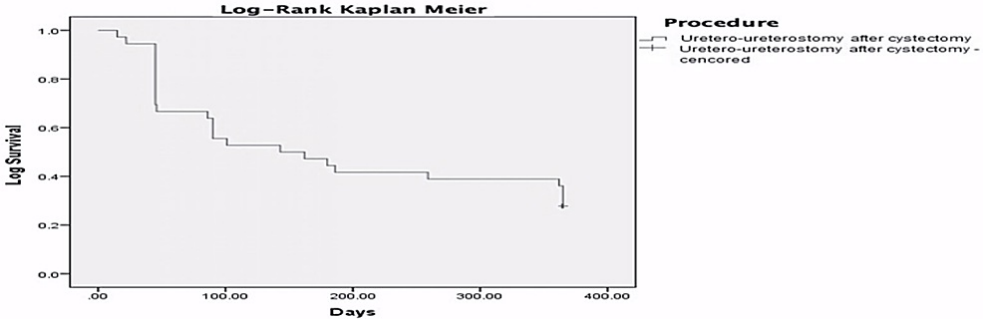
Absolute success - Kaplan Meier 365 days

**Figure 4:**
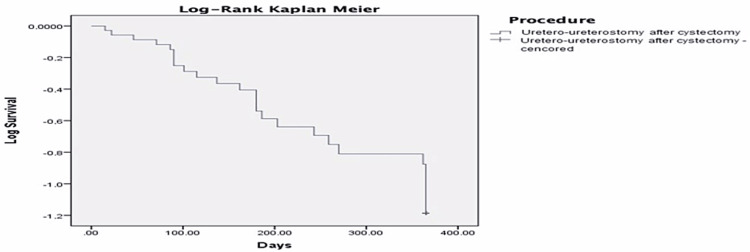
Relative success - Kaplan Meier 365 days

Summarizing the above results, the cumulative probability of absolute success was 97.2%, 69.4%, 61.1%, 50%, 33.3% at two weeks, six weeks, three months, six months and 12 months, respectively (Figure 3). Moreover, we observed a cumulative probability of qualified success in 97.2%, 91.7%, 72.2%, 58.3% and 41.7% at two weeks, six weeks, three months, six months and 12 months, respectively (Figure 4).

 

## Discussion

Both uretero-cutaneostomy and transuretero‑cutaneostomy are the most frequently performed types of urinary diversion without the use of intestinal segment and are generally reserved for frail and elderly patients [17-21]. Uretero-ureterostomy combined with unilateral nephrostomy is rarely performed; nonetheless, it has traditionally been used as a frequent type of urinary diversion in our Department [10-13]. This study confirms the previously reported favourable results of this method [12-15].

Renal function was found to remain relatively stable over the follow-up period, despite a non-significant change in the mean creatinine (17.5% increase, p=0.051) and mean eGFR (10.68% decrease, p=0.13) levels at the 12-month follow-up. Renal function was also stable in patients with postoperative pelvic dilatation. Oehler et al. reported uretero-ureterostomy with definite nephrostomy to have no impact on renal function in eight patients [12]. Other studies, however, have shown a continual deterioration of renal function following radical cystectomy. In comparison to our findings, Tombul et al. reported a higher loss of renal function within the first year (preop basal GFR: 70.68±18.47, first-year GFR: 53.96±18.48, p=0.161) in patients who underwent radical cystectomy and ureterocutaneostomy due to muscle-invasive BC [8]. In their retrospective observational study of 70 elderly patients with BC who underwent radical cystectomy, Creta et al. found eGFR to be constantly declining during follow-up (-26.5% after six months and -37.8% after 60 months) and suggested that these patients should be carefully informed about the risk of deterioration of renal function should they undergo surgery [22]. Similarly, Osawa et al. reported that 34.2% of patients undergoing cystectomy with four different types of urinary diversion, demonstrated reduced renal function during a median follow-up period of 34.5 months (12-228), with postoperative episodes of acute pyelonephritis and the presence of chemotherapy being significant adverse factors [23]. Data on the long-term preservation of renal function with transuretero‑cutaneostomy are currently lacking.

Acute pyelonephritis was diagnosed in eight patients (22.22%) postoperatively, although all patients were found to have a positive culture in urine collected from their nephrostomy tube. This incidence is in accordance with the reported in the literature for uretero-cutaneostomy and transuretero‑cutaneostomy (2.27%-42.30%) [24-27]. In six patients, acute pyelonephritis resulted from complications related to the nephrostomy tube (dislocation in three, obstruction and hydronephrosis in two, and post-replacement pyelonephritis in one). In the rest two cases, acute pyelonephritis was the result of reflux of infected urine into the contralateral kidney through the JJ-stent and occurred in the early postoperative phase with the JJ-stent in place.

Infected hydronephrosis was diagnosed in two patients. The first case was a random finding and the second patient suffered acute pyelonephritis and belongs to the above-mentioned eight patients of our cohort (Table 3). The condition was successfully treated with oral and intravenous antibiotics and increased fluid intake.

The overall rate of postoperative complications was low in our series. Although two patients died of complications related to surgery, these deaths were not associated with the uretero-ureterostomy per se. Furthermore, no skin strictures were noticed, as opposed to a 3.7%-75.9% stoma stricture rate occurring in patients with uretero-cutaneostomy [8,24,25,27]. However, a stenosis of the nephrostomy channel was found in four patients (11.11%) who, despite having their nephrostomy tube displaced, they presented with delay in the outpatients clinic. In these patients, nephrostomy had to be re-inserted under local anaesthesia. Overall, most patients had an uneventful postoperative course after ureteroureterostomy. Only two patients developed acute reflux pyelonephritis in the early postoperative phase. We summarize that bacteriuria in the nephrostomy-treated kidney was caused due to the presence of a JJ-stent that allowed reflux to reach the contralateral kidney, despite the administration of a single dose of ceftriaxone before cystectomy. Both cases were successfully managed with intravenous antibiotics.

Leakage from the anastomosis was noticed in two cases; the first was considered a failure in the technique of the anastomosis, as it occurred in the early postoperative period, whereas the other resulted from injury of the anastomosis during a subsequent sigmoidectomy for the treatment of obstructive ileus. Both cases were successfully treated with insertion of a second nephrostomy in the contralateral kidney. Furthermore, a ureteral JJ-stent was percutaneously inserted under sedoanalgesia in the above mentioned patient with the intraoperatively injured uretero-ureterostomy; both nephrostomy tubes were also left in place in this case. Khalilullah et al. reported stricture and leakage of the ureteric anastomosis in 4.5% of patients [19], slightly lower than the leakage in our study (5.6%) with no case of ureteral stricture. We assume that the sufficient blood perfusion of both ureters due to the less extended ureteral mobilization in comparison to the uretero-cutaneostomy leads to the absence of stenosis of the anastomosis.

Complications, including tube dislodgement, inability to remove the JJ-stent and anastomotic insufficiency were successfully treated by minimally invasive replacement of a nephrostomy under sedoanalgesia. No patient required a second open surgical procedure to treat postoperative complications. One patient refused ipsilateral exchange of the nephrostomy because of the discomfort he experienced in his preferred side-sleeping position. Thus, the placement of a nephrostomy in the contralateral kidney after tube obstruction was not considered to be a complication. The above-mentioned complications are considered as minor in comparison to the mentioned complications of the studies of Khalilullah and Wandschneider. Khalilullah et al. noted that 4.5% of their cohort underwent a re-operation due to a leaking ureteric anastomosis and 22.7% required JJ-stenting to treat stomal stricture [19]. Moreover, in a study by Wandschneider et al. open re-operations were required in seven patients (15.90%) with necrosis and stenosis of the urostomy. A JJ-stent was inserted as treatment of the stomal stricture in 11.36% of the sample [24]. Tombul et al. reported that 75.9% of the patients were treated with JJ-stent due to stomal stricture [8]. 

Limitations of our study include the small number of patients and the relatively short follow-up. In addition, this is a single-arm study and the comparison of the renal function with other types of urinary diversion could not be done. Further studies are needed to evaluate the long-term efficacy and safety of uretero-ureterostomy in combination with unilateral nephrostomy following radical cystectomy.

## Conclusions

This study demonstrated that uretero-ureterostomy combined with unilateral nephrostomy following radical cystectomy is a safe and effective method of urinary diversion for elderly patients with muscle-invasive BC who have a poor performance status. While larger, longer-term studies are needed to confirm these results, the absence of either severe surgical complications requiring re-operations or diversion-related deaths, as well as the satisfactory preservation of renal function indicate this type of urinary diversion to serve as a reliable alternative in this, difficult to treat, patient population.
